# Design and Optimization for 77 GHz Series-Fed Patch Array Antenna Based on Genetic Algorithm

**DOI:** 10.3390/s20113066

**Published:** 2020-05-28

**Authors:** Shuo Yang, Lijun Zhang, Jun Fu, Zhanqi Zheng, Xiaobin Zhang, Anmou Liao

**Affiliations:** 1 Institute of Microelectronics, Chinese Academy of Sciences, Beijing 100029, China; yangshuo@ime.ac.cn (S.Y.); zhanglijun@ime.ac.cn (L.Z.); zhengzhanqi@datangmobile.cn (Z.Z.); zhangxiaobin@ime.ac.cn (X.Z.); liaoanmou@ime.ac.cn (A.L.); 2 University of Chinese Academy of Sciences, Beijing 100049, China; 3 Institute of Microelectronics, Tsinghua University, Beijing 100084, China; 4 Beijing National Research Center for Information Science and Technology, Beijing 100084, China

**Keywords:** 77 GHz patch array antenna, millimeter-wave design, automotive radar, genetic algorithm

## Abstract

This paper proposes a method for designing a 77 GHz series-fed patch array antenna. Based on the traditional genetic algorithm, the study explores different array topologies consisting of the same microstrip patches to optimize the design. The main optimization goal is to reduce the maximum sidelobe level (SLL). A 77 GHz series-fed patch array antenna for automotive radar was simulated, fabricated, and measured by employing this method. The antenna length was limited to no longer than 3 cm, and the array only had a single compact series with the radiation patch about 1.54 mm wide. In the genetic algorithm used for optimization, the maximum sidelobe level was set equal to or less than −14 dB. The measurement results show that the gain of the proposed antenna was about 15.6 dBi, E-plane half-power beamwidth was about ±3.8°, maximum sidelobe level was about −14.8 dB, and H-plane half-power beamwidth was about ±30° at 77 GHz. The electromagnetic simulation and the measurement results show that the 77 GHz antenna designed with the proposed method has a better sidelobe suppression by over 4 dB than the traditional one of the same length in this paper.

## 1. Introduction

With the development of vehicular communication [1], as one of the most critical vehicle sensors, millimeter-wave automotive radars have attracted much research interest in recent years. Like most radars, automotive radars need to meet specific performance requirements [2]. As an essential part of radar, the antenna can determine the field of view, resolution, and detection distance. Different types of antennas have been designed for 77 GHz automotive radars. However, patch antennas are still the most popular ones [3,4,5] due to their low profile, low cost, and excellent compatibility with integrated circuits. A single patch antenna performs low-gain characteristic [6]. The patch antenna arrays [7,8] are always used in the 77 GHz automotive radars to meet the detection requirements, such as high gain, specified azimuth angle, elevation angle, and low maximum sidelobe level. 

At present, there are many kinds of design schemes for the patch array antennas used in 77 GHz automotive radars. The 77 GHz microstrip patch antenna arrays in [9,10] are designed to traditional series-fed type. The same patches are arranged in a string separated by an electrical wavelength. This traditional design method is simple, but it is not an excellent design method. To optimize the sidelobe suppression of the patch array antenna in the case of finite array elements, many designers prefer to design the 77 GHz patch array antenna in different tapered distributions, such as Dolph-Chebyshev distribution or Taylor distribution, to improve the antenna array sidelobe suppression [11,12,13]. Theoretically, they are great design solutions. However, due to the different sizes of the patches, the requirements of manufacturing resolution and accuracy are relatively high. Moreover, some works [14,15,16] offer 77 GHz antenna designs that can improve the overall directivity of the antenna by combining the patch or the patch arrays with the lens. However, an extra lens surely adds design cost and energy loss. While reaching gain, angles, and sidelobe level (SLL) requirements, the 77 GHz automotive radar antenna also needs to be designed as compact as possible [17]. In [18], a high-gain low sidelobe level 77 GHz patch array antenna is proposed. However, this antenna is difficult to implement, for its size and the process precision are not taken into consideration. The paper [19] proposes a compact patch array antenna for 77 GHz radar with a novel power divider design. However, due to the non-uniformity of the current distribution, the meander divider lines inevitably bring unwanted radiation. Different from the others, the present work proposes an optimization method from the perspective of array factors and focuses on analyzing the influence of the array topologies on SLL of the series-fed patch array antenna. A simple genetic algorithm was employed to explore the possible limitation of array topologies to the optimization of SLL, avoiding complex manual calculations. Additionally, this paper proposes a new optimization method of the series-fed patch array antenna. Unlike the genetic algorithms [20,21,22,23,24] generally set for antenna designs, the genetic algorithm’s genes in this paper represent different array factors rather than patch amplitudes or phases. Finally, a 77 GHz series-fed patch array antenna with high gain and low sidelobe was designed based on this optimization method. Compared with the traditional simple series fed array, it has better sidelobe suppression. Compared with tapered distributions, it has low profile characteristics. The low-cost material Rogers5880 was selected for simulation and manufacture. The final antenna can meet the 77 GHz vehicle radar antenna’s requirements without an additional lens focusing beam.

The main contents of this paper are structured as follows. Section 2 describes the beam synthesis theory of the *N*-element linear array antenna, which is well known by most antenna designers, and it is applied in our genetic algorithm. Section 3 makes a process introduction to the combination of the design and the genetic algorithm. Simulation and measurement results of the specially designed and optimized series-fed 77 GHz patch array antenna and the traditional counterpart are shown comparatively in Section 4. Finally, some conclusions are given in Section 5.

## 2. Beam Synthesis Theory of the N-Element Linear Array Antenna

**Figure 1:**
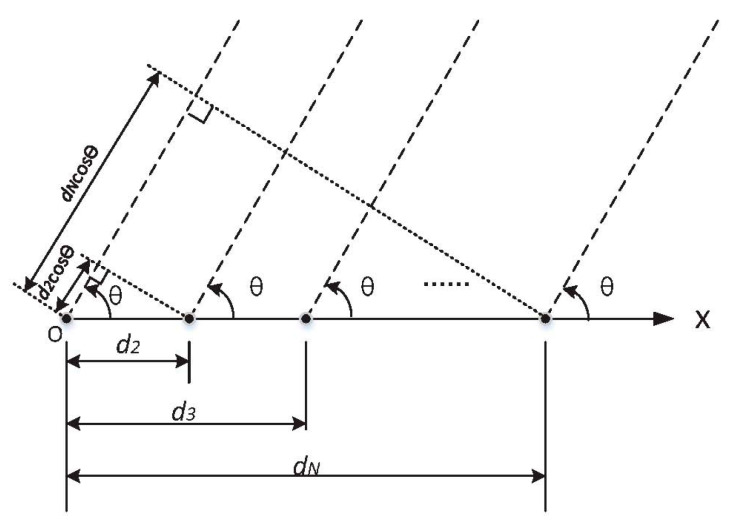
Schematic diagram illustrating the beam synthesis of the *N*-element linear array antenna.

A schematic diagram illustrating the beam synthesis of *N*-element linear array antenna is shown in Figure 1, where the solid black dots represent the center of each antenna element in the linear array, *d_n_* (*n* = 1, 2, 3 … *N*) represents the distance between the center of the element *n* and the reference origin O,. and *N* is the total number of similar elements in the array. Suppose that the radiation pattern of the element is *F_0_*(*θ, ϕ*), and the feeding current to the n-th element is ***I****_n_* (*n* = 1, 2, 3… *N*). According to Figure 1, the radiation pattern of the linear array [25] is expressed as:

(1) $$f\left(\theta ,\;\phi \right)=F_{0}\left(\theta ,\;\phi \right)\overset{N}{\underset{n=1}{\sum }}I_{n}e^{jkd_{n}\cos\left(\theta \right)}$$

Assume that the amplitude of the element *n* is *m_n_*, and the element *n* has a progressive phase lead *β_n_* current excitation relative to the first element (*O*). Then, the array factor can be given by:

(2) $$AF=\overset{N}{\underset{n=1}{\sum }}I_{n}e^{jkd_{n}\cos\left(\theta \right)}=\overset{N}{\underset{n=1}{\sum }}m_{n}e^{j\beta_{n}}e^{jkd_{n}\cos\left(\theta \right)}$$

## 3. Design Method Description

After introducing the algorithm flow in Section 3.1, this paper then takes the proposed series-fed patch array antenna for 77 GHz medium-range automotive radar in Section 3.2 to illustrate the design method.

### 3.1. Description of the Genetic Algorithm Flow

The necessary steps of the genetic algorithm used in our design method are as follows:

Step 1. The element radiation pattern is obtained from High Frequency Structure Simulator (HFSS) electromagnetic simulation, and the simulated data are then stored into matrix *F* for later use.

Step 2. Generate the initial population. In this step, the individuals are completely randomly generated within the range of *N* bits. A matrix named *M* is used to represent an individual, and each individual is a random binary code. 

Step 3. Based on the beam synthesis theory shown in Figure 1 multiplying the element radiation pattern (matrix *F*) by random array factors produced by the computer, overall random radiation patterns can be obtained. However, it cannot guarantee that individuals must meet the specified requirements in the initial population. If no individuals are meeting the requirements, the algorithm flow goes back to the beginning of Step 2.

Step 4. Selection, crossover, and mutation.

**Figure 2:**
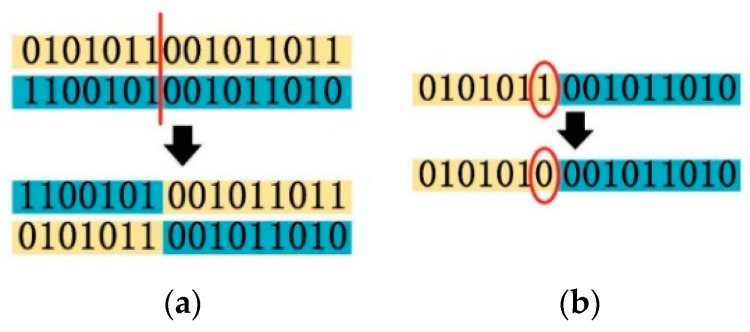
(**a**) Crossover process; (**b**) mutation process.

The crossover and the mutation methods are similar to the traditional genetic algorithm [26,27,28], as shown in Figure 2a,b, respectively. 

Step 5. The algorithm is executed to the maximal number of generations we set. Then, we select the best individual from the final population as the approximate global optimal solution. The best individual in this paper is the one with the best SLL.

Software MATLAB was used for coding and data processing to finish the above processes. It should be noted that not every result converges to the same one in the above process. The optimization scheme we finally chose is the one with a probability of more than 90% in the genetic algorithm. When setting the mutation probability to 10%, the crossover probability to 80%, and the maximal generation number to 50, the running time of MATLAB under an Intel Core i7 computer was less than 20 s.

### 3.2. Design of the 77 GHz Series-Fed Patch Array Antenna

In this part, a 77 GHz series-fed patch array antenna was designed based on the genetic algorithm flow introduced in Section 3.1. Before designing the 77 GHz antenna, we chose Rogers5880 as the substrate (*ε_r_* = 2.2, the thickness = 254 µm).

**Figure 3:**
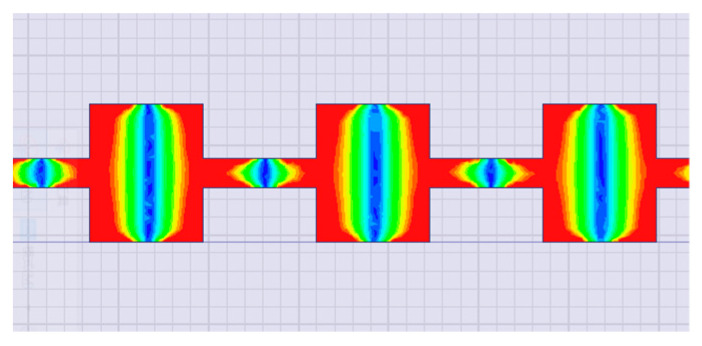
Schematic diagram of electric field distribution of a microstrip patch antenna array.

**Figure 4:**
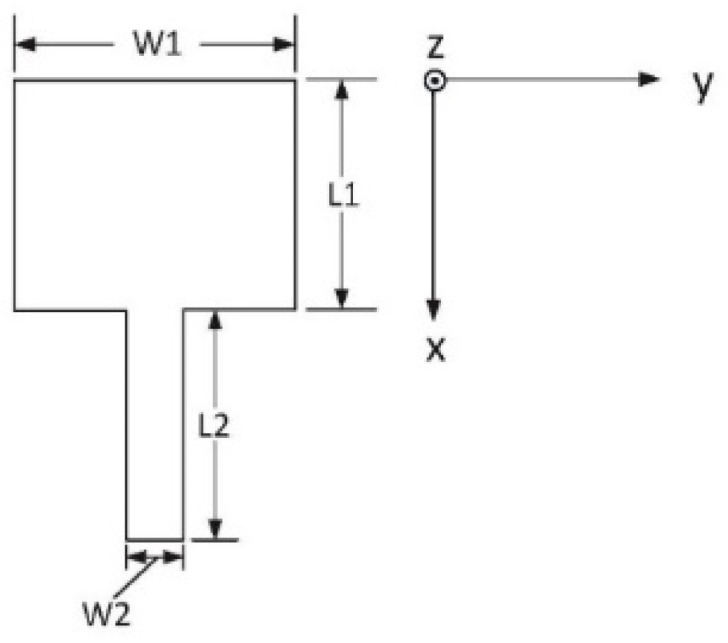
Schematic diagram of a microstrip patch antenna.

The approximate size of the patch was calculated by using the traditional empirical formulas [29] and then finely tuning the length of the patch in HFSS until the electric field distributed symmetrically on the patch at 0° source signal (77 GHz sine signal), as shown in Figure 3. The final main parameters of the patch used in this paper were as follows: L_1_ = 1260 µm, W_1_ = 1540 µm, L_2_ = 1260 µm, and W_2_ = 320 µm. Then, the radiation pattern of the antenna element shown in Figure 4 was extracted from HFSS simulation, and we selected *E*-plane radiation intensity data from HFSS simulation and stored them in matrix *F* = [*F*_1_, *F*_2_, …, *F_r_*,… *F*_360_], where *F_r_* (*r* = 1, 2, 3 …, 360) represented the intensity of the far-field electric field when theta was degree *r*.

**Figure 5:**
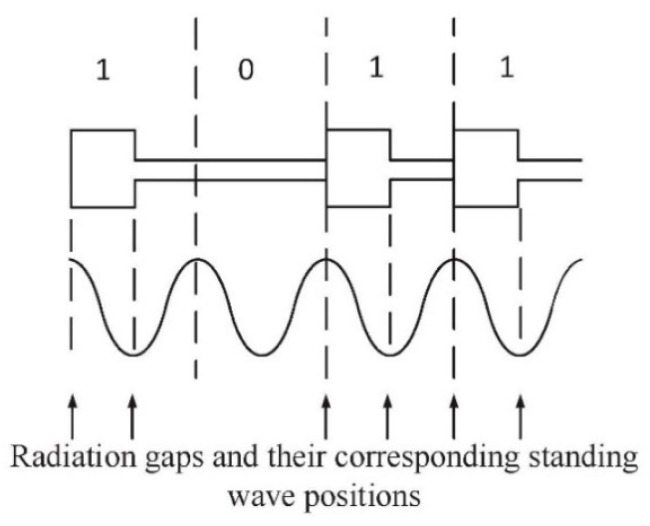
The topology structure corresponding to the code “1011”.

In the genetic algorithm applied in the design method, multiple sets of binary sequences were randomly generated to represent the population (matrix *M*). The "1" in our algorithm represented a radiation patch and its feeder, and “0” meant that there was no radiation patch and it was to be replaced by a transmission line of one-wave electrical length at 77 GHz. For example, the corresponding topology structure of code “1011” is shown in Figure 5. Note that the amplitude of each element of the series-fed array was assumed to be equal, and the radiation of the transmission line was ignored in the genetic algorithm. 

As shown in Figure 5, we made all the radiating slots of the rectangular patch at the position of the oscillation antinodes of the standing wave. Therefore, the total length of the radiant patch and its feeder, which “1” represents, should have been one wave in electrical length at 77 GHz as well as for the transmission line, which “0” stands for. In this case, letting the array factor be *AF*_ga_, from the Equation (2), we get *AF_ga_* as:

(3) $$AF_{ga}=\overset{N}{\underset{n=1}{\sum }}m_{n}e^{j\beta_{n}}e^{jkd_{n}\cos\left(\theta \right)}=\overset{N}{\underset{n=1}{\sum }}m_{n}e^{j\left(n-1\right)2\pi }e^{jkd_{n}\cos\left(\theta \right)}=\overset{N}{\underset{n=1}{\sum }}m_{n}e^{jkd_{n}\cos\left(\theta \right)}$$

Corresponding to Figure 5, *N* = 4, *m*_1_ = 1, *m*_2_ = 0, *m*_3_ = 1, and *m*_4_ = 1. The genetic algorithm randomly generates multiple array factors. Then, the array antenna radiation patterns with the same array elements and different array factors are calculated.

According to the Euler formula *e^jx^=cos(x)+jsin(x),* the array factor in Equation (3) can be expressed as:

(4) $$AF_{ga}=\overset{N}{\underset{n=1}{\sum }}m_{n}e^{jkd_{n}\cos\left(\theta \right)}=\overset{N}{\underset{n=1}{\sum }}m_{n}\left[\cos\left(kd_{n}\cos\left(\theta \right)\right)+jsin\left(kd_{n}\cos\left(\theta \right)\right)\right]$$

Then, the magnitude of *AF_ga_* can be expressed as:

(5) $$|AF_{ga}|=\sqrt{[\;\overset{N}{\underset{n=1}{\sum }}m_{n}\cos\left(kd_{n}\cos\left(\theta \right)\right){]}^{2}+[\overset{N}{\underset{n=1}{\sum }}m_{n}\sin\left(kd_{n}\cos\left(\theta \right)\right){]}^{2}}$$

Corresponding to the Rogers5880-based 77 GHz patch in Figure 4, we get that $k=\frac{2\pi }{\lambda_{0}}=\frac{2\pi f}{c}\approx \frac{2\times 3.14\times 77\times 10^{9}\;\mathrm{Hz}}{3\times 10^{8}\;{\mathrm{ms}}^{-1}}\approx 1611.87\;m^{-1}$ and the physical length as *d*_*n*+1_ − *d_n_* = L_1_ + L_2_ = 2520 µm (Figure 4). Assuming that *d*_0_ = *d*_*n*+1_ − *d_n_* = 2520 µm, from Equation (5), we get:

(6) $$\begin{array}{l} |AF_{ga}|=\sqrt{[\;\overset{N}{\underset{n=1}{\sum }}m_{n}\cos\left(k\left(n-1\right)d_{0}\cos\left(\theta \right)\right){]}^{2}+[\overset{N}{\underset{n=1}{\sum }}m_{n}\sin\left(k\left(n-1\right)d_{0}\cos\left(\theta \right)\right){]}^{2}}\\ =\sqrt{[\overset{N}{\underset{n=1}{\sum }}m_{n}\cos\left(4.062\times \left(n-1\right)\cos\left(\theta \right)\right){]}^{2}+[\overset{N}{\underset{n=1}{\sum }}m_{n}\sin\left(4.062\times \left(n-1\right)\cos\left(\theta \right)\right){]}^{2}} \end{array}$$

MATLAB coding was used to generate multiple *N*-bit binary matrices as genes randomly. The term of every gene was *M* = [*m*_1_, *m*_2_, *m*_3_… *m_N_*] with the same number of elements as the expected antenna array. Multiple *M* matrices generated randomly were substituted into Equation (5) to obtain multiple genetic array factors (|*AF_ga_* |). In order to multiply with the element radiation pattern matrix *F*, we also put 360 theta values (*θ* ranges from 1° to 360°) into the Equation (5) and obtained multiple matrices such as *F_ga_* = [*F_ga1_*, *F_ga2_*_,_ … *F_gar_*,…, *F_ga360_*], where *F_gar_* (*r* = 1,2,3…360) represents the magnitude of |*AF_ga_*| in Equation (5) when theta is degree r. This meant that multiple *AF_ga_* matrices were also random because the *M* matrices representing the genes were random. Additionally, the number of random *F_ga_* matrices in each generation was equal to the number of the population set by the designer.

According to Equation (1), we multiplied the same position values in the *F* matrix by *F_ga_* matrices and obtained new matrices similar to *F_al_* = [*F_al1_*, …, *F_alr_*, …, *F_al360_*] = [*F_1_F_ga1_*, …, *F_r_F_gar_*, …, *F_360_F_ga360_*] (*r* = 1,2,3…360). In the genetic algorithm, the matrix *F_al_* represents the E-plane radiation diagram of the random patch antenna arrays.

Finally, the genetic algorithm dealt with multiple *F_al_* matrices. The filter algorithm in this paper mainly considered the SLL. The filter function is as follows:

(7) $$\frac{F_{al90}}{F_{al\theta }}>10^{\frac{SLL_{goal}}{20}}\;\left(\theta \;\in \;\left[1,90-\theta_{goal}]\;\cup \;[90+\theta_{goal},360\right]\right)$$

For a 77 GHz automotive radar, we set the *E*-plane first null beamwidth within ±8° (*θ_goal_* = 8 in Inequality (7)), and the sidelobe suppression goal was set to 14 dB (*SLL_goal_* in Inequality (7)). The optimized value was obtained after generations of selection, crossover, and mutation through the loop calculation.

Considering the practical application, the 77 GHz automotive radars are very small with a scale of several centimeters, thus the length of the gene in this paper was limited to 10 bits. Finally, the optimal individual was selected as “1011111111” when there were 10 fixed positions in the array.

As mentioned before, by using the electromagnetic simulation tool, the phases of each patch in the serial array could be ensured to be synchronized by fine-tuning the size of the patch. However, considering practical problems such as mutual coupling or element matching degradation, the radiation amplitudes of patch elements in different positions were different. Our proposed genetic algorithm ignored the amplitude difference of patches at different positions. Electromagnetic simulation or measurement verification was needed to verify the usability of the proposed topology from the genetic algorithm.

## 4. Electromagnetic Simulation and Measurement Verification

**Figure 6:**
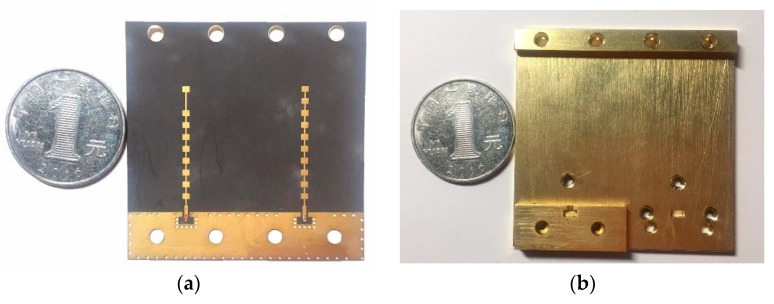
(**a**) The 77 GHz patch array antennas; (**b**) the copper components for measurement.

In Figure 6a, the left antenna corresponds to the “1011111111” design code introduced in Section 3.2, and the antenna on the right is “1111111111”, which indicates a traditional structure. In addition, due to the high frequency of 77 GHz, the WR-12 waveguide was used to keep compatible with the measurement instruments. The auxiliary components for measurement included a quarter-wavelength shorted WR-12 waveguide for the reflection and a WR-12 waveguide for the signal transmission. The copper components for waveguide-microstrip transition are shown in Figure 6b. The coin in Figure 6 is a Chinese one-yuan coin with a diameter of 25 mm. 

**Figure 7:**
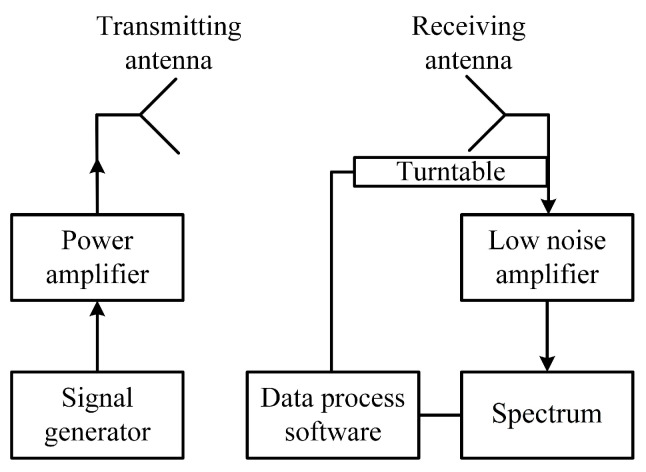
Simplified diagram of the measurement framework.

As shown in Figure 7, the signal generator (N5183A) was set to the point-frequency mode (77 GHz). The receiving antenna is a known horn antenna. The model of the low noise amplifier is ELNA-7579-25, and the spectrum analyzer model is E4446A. The antenna gain measurement in this paper adopted the comparative method. First, we adjusted the angle of the antenna being measured to make the receiving power reach the maximum level *P*_x_ (dBm). Then, we replaced the transmitting antenna with a standard horn antenna whose gain value is known as *G*_s_ (dBi) and adjusted this standard horn antenna angle; thus, the receiving maximum power level *P*_s_ (dBm) was obtained. Assuming that the gain of the antenna under measurement was *G*_x_, we obtained the gain of the antenna to be measured as:

(8) $$G_{x}=G_{s}-(P_{s}-P_{x})$$

**Figure 8:**
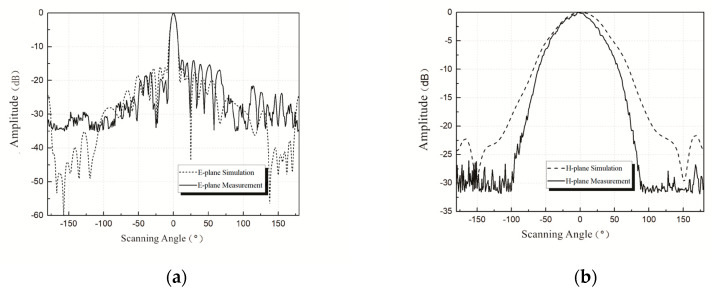
(**a**) Simulation and measurement results on *E*-plane of “1011111111” patch array antenna; (**b**) simulation and measurement results on *H*-plane of “1011111111” patch array antenna.

Figure 8 shows the simulation and the measurement results of the normalized radiation pattern for the series-fed antenna array “1011111111”. The HFSS simulation results show that series-fed array antenna gain was about 17 dBi at 77 GHz, *E*-plane half-power beamwidth was about ±3.7°, SLL was about −16.5 dB, and *H*-plane half-power beamwidth was about ±36°. The measurement results show that, at 77 GHz, antenna gain was about 15.6 dBi, *E*-plane half-power beamwidth was about ±3.8°, SLL was about −14.8 dB, and *H*-plane half-power beamwidth was about ±30°.

**Figure 9:**
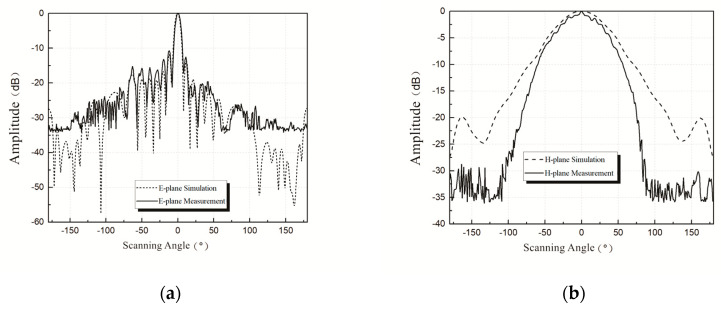
(**a**) Simulation and measurement results on *E*-plane of “1111111111” patch array antenna; (**b**) simulation and measurement results on *H*-plane of “1111111111” patch array antenna.

Figure 9 is similar to Figure 8 but for the “1111111111” patch array antenna. Simulation results show that the gain of the antenna corresponding to “1111111111” was about 17 dBi at 77 GHz, *E*-plane half-power beamwidth and SLL were about ±3.7° and −12.5 dB, respectively, and *H*-plane half-power beamwidth was about ±33°. The measurement results show that, at 77 GHz, antenna gain was about 15.9 dBi, *E*-plane half-power beamwidth was about ±3.7°, SLL was about −10 dB, and *H*-plane half-power beamwidth was about ±30°.

Simulation and measurement results both show that the “1011111111” antenna has a better sidelobe suppression of *E*-plane than that of the “1111111111” antenna. The gain and the half-power beamwidth of the two antennas meet the design requirements.

It is shown in Figure 8 and Figure 9 that the simulation results of the 77 GHz antenna were found to deviate somewhat from the measurement results. The authors believe that there are several main reasons for deviations. On the one hand, there exist differences between simulation and reality, and manufacturing tolerances or measurement errors inevitably affect the experimental results. On the other hand, the addition of auxiliary components for measurement could also affect the antenna. Moreover, the Rogers5880 substrate used here has the characteristics of softness, thinness, and deformability, which may also add unexpected uncertainties.

## 5. Conclusions

This paper proposed a new method for designing a 77 GHz series-fed patch array antenna for automotive radar based on a simple genetic algorithm. The proposed 77 GHz patch array antenna and a traditional simple 77 GHz patch array antenna with the same size were designed, made, and measured. Electromagnetic simulation and measurement verification were implemented for the two antennas. The electromagnetic simulation and measurement results showed that, compared with the simple series-fed design, the 77 GHz patch array antenna based on the new design scheme had a better sidelobe suppression by over 4 dB at 77 GHz than the simple one.
